# Association between Vitamin D Supplementation and Cancer Mortality: A Systematic Review and Meta-Analysis

**DOI:** 10.3390/cancers14153717

**Published:** 2022-07-30

**Authors:** Renjie Zhang, Yu Zhang, Zheran Liu, Yiyan Pei, Ping Xu, Weelic Chong, Yang Hai, Ling He, Yan He, Jiayi Yu, Jingjing Wang, Fang Fang, Xingchen Peng

**Affiliations:** 1 Department of Biotherapy, Department of Neurosurgery, West China Hospital, Sichuan University, Chengdu 610041, China; zrjwch@stu.scu.edu.cn; 2 Evidence-Based Medicine Center, Affiliated Hospital of Chengdu University, Chengdu 610084, China; zhangyu1057@cdu.edu.cn; 3 Department of Biotherapy and National Clinical Research Center for Geriatrics, Cancer Center, West China Hospital, Sichuan University, Chengdu 610041, China; liuzheran@stu.scu.edu.cn (Z.L.); rocky@stu.scu.edu.cn (Y.P.); heling1@stu.scu.edu.cn (L.H.); heyan1@stu.scu.edu.cn (Y.H.); 2020324025318@stu.scu.edu.cn (J.W.); 4 Sichuan University Library, Sichuan University, Chengdu 610047, China; xuping@scu.edu.cn; 5 Department of Medical Oncology, Thomas Jefferson University, Philadelphia, PA 19144, USA; wxc026@jefferson.edu; 6 Sidney Kimmel Medical College, Thomas Jefferson University, Philadelphia, PA 19144, USA; yang.hai@jefferson.edu; 7 School of Medical and Life Sciences, Chengdu University of Traditional Chinese Medicine, Chengdu 611137, China; yujiayi@stu.cdutcm.edu.cn; 8 Department of Neurosurgery, West China Hospital, Sichuan University, Chengdu 610041, China; fangfang01@scu.edu.cn

**Keywords:** Vitamin D supplementation, cancer mortality, cancer incidence, meta-analysis

## Abstract

**Simple Summary:**

It has been questioned whether vitamin D supplements can reduce the mortality and incidence of tumors. In this systematic review and meta-analysis of 12 randomized controlled trials with a total of 72,669 participants, vitamin D supplementation could not reduce the cancer mortality or cancer incidence. Our results suggest a reconsideration of the previous view that vitamin D supplementation could reduce overall cancer mortality is needed.

**Abstract:**

Background: Vitamin D deficiency is related to increased cancer risk and deaths. However, whether vitamin D supplementation reduces cancer mortality remains unclear, and several randomized controlled trials yield inconsistent results. Methods: Medline, Embase, and the Cochrane Central Register of Controlled Trials were searched from their inception until 28 June 2022, for randomized controlled trials investigating vitamin D supplementation. Pooled relative risks (RRs) and their 95% confidence intervals (CIs) were estimated. Trials with vitamin D supplementation combined with calcium supplementation versus placebo alone and recruiting participants with cancer at baseline were excluded in the present study. Results: This study included 12 trials with a total of 72,669 participants. Vitamin D supplementation did not reduce overall cancer mortality (RR 0.96, 95% CI 0.80–1.16). However, vitamin D supplementation was associated with a reduction in lung cancer mortality (RR 0.63, 95% CI 0.45–0.90). Conclusions: Vitamin D supplementation could not reduce cancer mortality in this highly purified meta-analysis. Further RCTs that evaluate the association between vitamin D supplementation and total cancer mortality are still needed.

## 1. Introduction

In recent years, supplementation with vitamin D has been viewed as a potential strategy for preventing cancer [1,2,3]. Evidence from observational, preclinical, and clinical studies strongly suggests that low 25-hydroxyvitamin D [25(OH)D] status is associated with the risk of developing colorectal cancer [4], breast cancer [5,6], bladder cancer [7,8], lung cancer [9,10], pediatric cancer [11], pancreatic cancer [12], and prostate cancer [13]. If adequate vitamin D concentrations reduce cancer risk, vitamin D supplementation may be a readily available, safe, and economical modality to reduce cancer incidence and mortality [2]. However, randomized controlled trials (RCTs) testing Vitamin D supplementation have been inconsistent, with one study finding that the incidence of cancer is reduced, while the other concluding that cancer mortality remains unchanged [14,15].

Previous systematic reviews found that vitamin D supplementation reduced cancer mortality [16,17,18,19,20]. However, these studies lacked enough detail on the associations for site-specific cancers and have not evaluated the quality of evidence using the Grading of Recommendations Assessment, Development and Evaluation (GRADE) and an estimation of optimum sample size using trial sequential analyses (TSA). Since this study, the results of a new large randomized trial, the D-Health trial, changed the landscape of evidence, which suggested a trend of an increase in cancer mortality (hazard ratios 1.15, 95% CI 0.96 to 1.39) in a Vitamin D-replete Australian population.

Therefore, we performed a systematic review, meta-analysis, and trial sequential analyses to summarize the most recent evidence and assess the effect of vitamin D supplementation on cancer mortality.

## 2. Materials and Methods

### 2.1. Protocol and Guidance

We followed the Preferred Reporting Items for Systematic Reviews and Meta-Analyses (PRISMA) guidelines for reporting our systematic review [21]. This study was conducted according to the protocol registered in the PROSPERO database (CRD42019119639).

### 2.2. Eligibility Criteria

Studies that met the following criteria were included: (1) Population: adults (age ≥ 18) with any health condition; (2) Intervention: vitamin D supplements at any dose and for any duration. Trials of vitamin D plus calcium vs. calcium alone were considered vitamin D interventions; (3) Comparison intervention: placebo or no treatment. If other interventions were given (e.g., calcium), they had to be the same in all groups; (4) Outcome: cancer mortality or cancer incidence, with a follow-up of more than one year. The primary outcome was overall cancer mortality. Secondary outcomes were overall cancer incidence, site-specific cancer mortality, and incidence (i.e., breast, lung, prostate, colorectal). (5) Study design: randomized controlled trials (RCT), including quasi-randomized and cluster-randomized.

Studies were excluded if they were (1) case reports, case series, and observational studies, (2) trials of hydroxylated vitamin D or vitamin D analogs, (3) trials where all participants received vitamin D, (4) trials where all participants have cancer, (5) trials of pregnant or lactating women, (6) trials of critically ill patients, (7) trials with the total number of an outcome less than ten because of the small effect size and/or short follow-up time [16], (8) trials with vitamin D supplementation combined with calcium supplementation versus placebo alone because evidence showed calcium supplementation was associated with other unfavorable effects, including mortality [22], cardiovascular (e.g., myocardial infarction) [23,24,25], and breast cancer risk [26].

### 2.3. Data Sources and Search Strategy

An experienced research librarian (PX) developed and executed the search strategy. The electronic databases Medline, Embase, and Cochrane Central Register of Controlled Trials were searched (Table S1). We also checked the reference lists of eligible studies as well as screened scientific abstracts and relevant clinical trial registries (ClinicalTrials.gov and the World Health Organization International Clinical Trials Registry Platform). The last electronic search was performed on 28 June 2022. There were no restrictions on language.

### 2.4. Study Selection and Collection

Eight investigators were divided into two groups independently, and in duplicate screened the titles and abstracts of all identified studies using a priori selection criteria. They screened the full text of potentially relevant studies. Disagreements were resolved by discussion or, if needed, by consensus. Then, data were extracted from the included RCTs using a purpose-built spreadsheet containing the following information: Author names, publication years, the interventions in each arm, the number of total participants and events in each arm, baseline circulating 25(OH)D levels, primary outcome, and the follow-up time.

### 2.5. Assessment of Risk of Bias and Quality of Evidence

Two investigators independently performed quality assessments. The Cochrane risk of bias assessment tool was used to assess the risk of bias among the eligible trials. The quality assessment took random sequence generation, allocation concealment, blinding of participants, staff, and outcome assessors; incomplete outcome data; selective outcome reporting; and other potential biases into account. The risk of bias for each domain was graded as high, low, or unknown. The overall risk of bias for the study was reflected by the highest risk of bias for any criteria.

We used the Grading of Recommendation, Assessment, Development, and Evaluation (GRADE) approach (GRADE Pro-version 3.6 software) to generate the absolute and relative risk of the outcomes [27]. The GRADE guidance rated the quality of evidence and strength of recommendations depending on study design limitations, inconsistency, indirectness, publication bias, and imprecision in each result.

### 2.6. Data Synthesis and Analysis

The meta-analysis for the included studies were conducted using Review Manager (RevMan, version 5.4.1, the Nordic Cochrane Center, the Cochrane Collaboration) and the metafor package in R (version 4.0.1; R Project for Statistical Computing). All analyses were based on the intention-to-treat approach. The meta-analysis was conducted using random-effect models regardless of the level of heterogeneity. The risk ratio (RR) and 95% confidence intervals (CI) were calculated for dichotomous data. All tests of statistical inference reflect a 2-sided of *p* < 0.05. Statistical heterogeneity of the data was assessed by using the I^2^ test [28]. We directly applied the random-effect models to our meta-analysis, considering the potential inconsistency in the included studies. If there are more than ten RCTs in a meta-analysis, publication bias was assessed by funnel plot techniques and the Egger and Begg tests.

Trial sequential analysis (TSA) was used to evaluate the statistical reliability of the pooled results and adjust for the random error risk using TSA software (version 0.9.5.10, beta) [29]. When the cumulative Z-curve entered the futility area or crossed the trial sequential monitoring boundaries, it suggested the anticipated intervention effect was sufficient and conclusive; thus, no further trials were needed. We applied TSA to keep an overall 5% risk of type I error and 80% power, assuming the intervention effect could reduce 20% relative risk.

Subgroup analyses were performed according to baseline vitamin D status (insufficiency and adequacy), type of vitamin D (vitamin D2 and vitamin D3), dose (≥2000 IU/d and <2000 IU/d), the dosing frequency of treatment (daily and intermittently), length of follow-up (≥3 years and <3 years), treatment duration (≥3 years and <3 years), and co-therapy status (without calcium and with calcium). We conducted post-hoc subgroup analyses based on the number of patients (≥2000 and <2000), number of events (≥200 and <200), mean age (≥70 years and <70 years), sex (female and both), and published year (before 2014 and in or after 2014).

Sensitivity analyses were conducted by (1) excluding trials with high or unknown risk of bias, (2) excluding trials with a high risk of bias of each domain, (3) excluding quasi-randomized or cluster-randomized trials, (4) excluding the largest trial, and (5) using fixed-effect models.

## 3. Results

### 3.1. Characteristics of Included Studies

**Figure 1:**
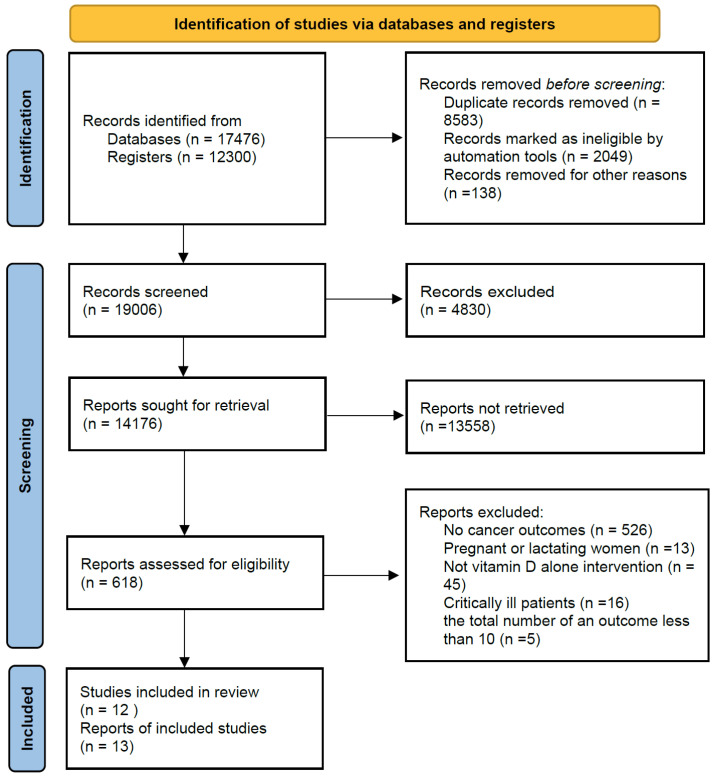
Flowchart for study selection.

After identifying 29,776 articles, a total of 12 RCTs met the inclusion criteria (Figure 1) [14,15,30,31,32,33,34,35,36,37,38,39,40,41,42,43,44]. Characteristics of included studies are present in Table 1. Among these RCTs, 5 RCTs were conducted in Europe, 4 RCTs were in the United States, and 2 RCTs were in Australia, and 1 in New Zealand. Two RCTs only included female participants, while others included male and female participants. Mean circulating levels of 25(OH)D for the vitamin D supplementation group and placebo group ranged from 38 to 77 nmol/L.

Included RCTs were generally at low or unclear risk of bias. Risk-of-bias assessments are reported in Figures S1 and S2. Of the 12 included trials, 5 were low risk of bias, 6 were unclear risk, and 1 was high risk.

### 3.2. Cancer Mortality

**Figure 2:**
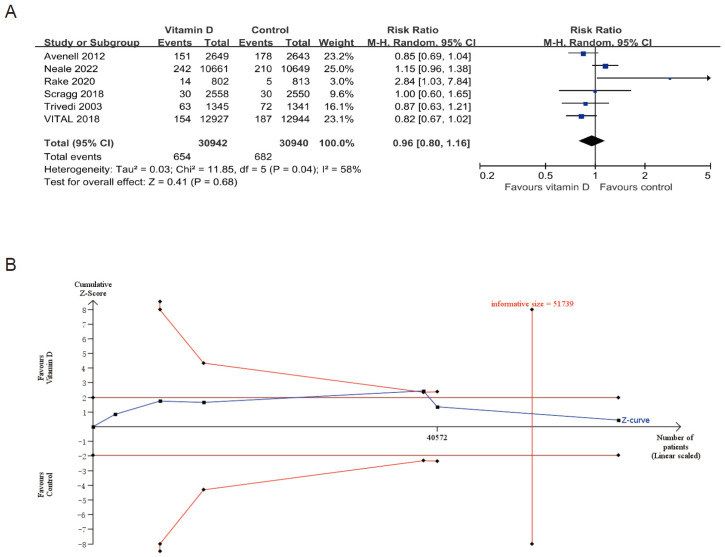
Exploring the relationship between vitamin D supplementation and cancer mortality. (**A**) the forest plot. (**B**) The plot of trial sequential analysis. Trial sequential monitoring boundaries were not crossed by the cumulative Z-score curve and with the addition of the latest trial, the pooled sample size exceeded the required information size.

Of these, 6 RCTs with a total of 61,882 participants were included in the meta-analysis for cancer mortality [15,37,38,40,41,45]. Pooled RR showed that vitamin D supplementation did not reduce cancer mortality risk (RR 0.96, 95% CI: 0.80–1.16, I^2^ = 58%; Figure 2A). TSA analyses of cancer mortality showed that future trials are unlikely to change the pooled estimate (Figure 2B) [15]. The funnel plot revealed no evidence of publication bias for the overall cancer mortality (Figure S3).

**Figure 3:**
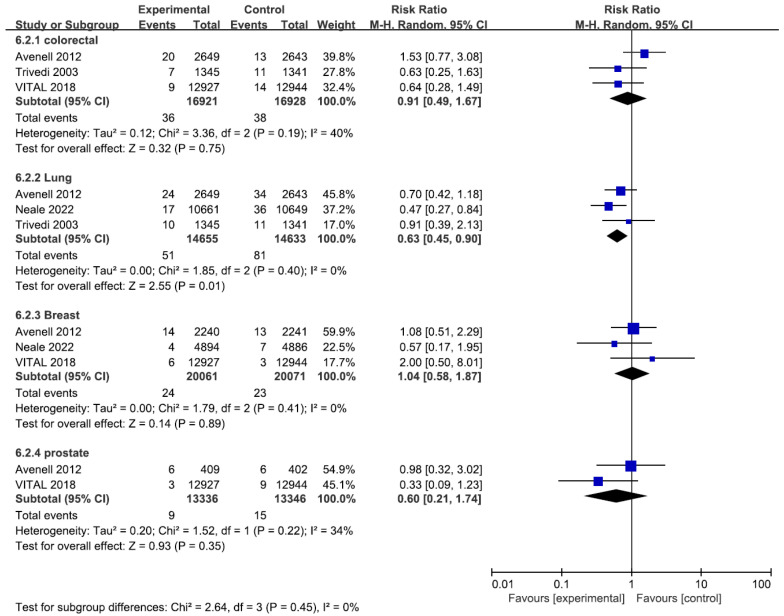
The forest plot of vitamin D supplementation and site-specific cancer mortality.

Subgroup analyses demonstrated that only participants with daily dosing vitamin D have lower cancer mortality compared with those dosing vitamin D intermittently (RR 0.84, 95% CI 0.72–0.97, Table S2). All sensitivity analyses on cancer mortality were consistent with the main analyses, demonstrating vitamin D supplementation did not reduce cancer mortality (Table S3). For the site-specific cancer mortality, vitamin D supplementation significantly reduced lung cancer mortality (RR 0.63, 95% CI: 0.45–0.90, I^2^ = 0%) while the results of other outcomes were consistent with the overall cancer mortality (Figure 3).

### 3.3. Cancer Incidence

**Figure 4:**
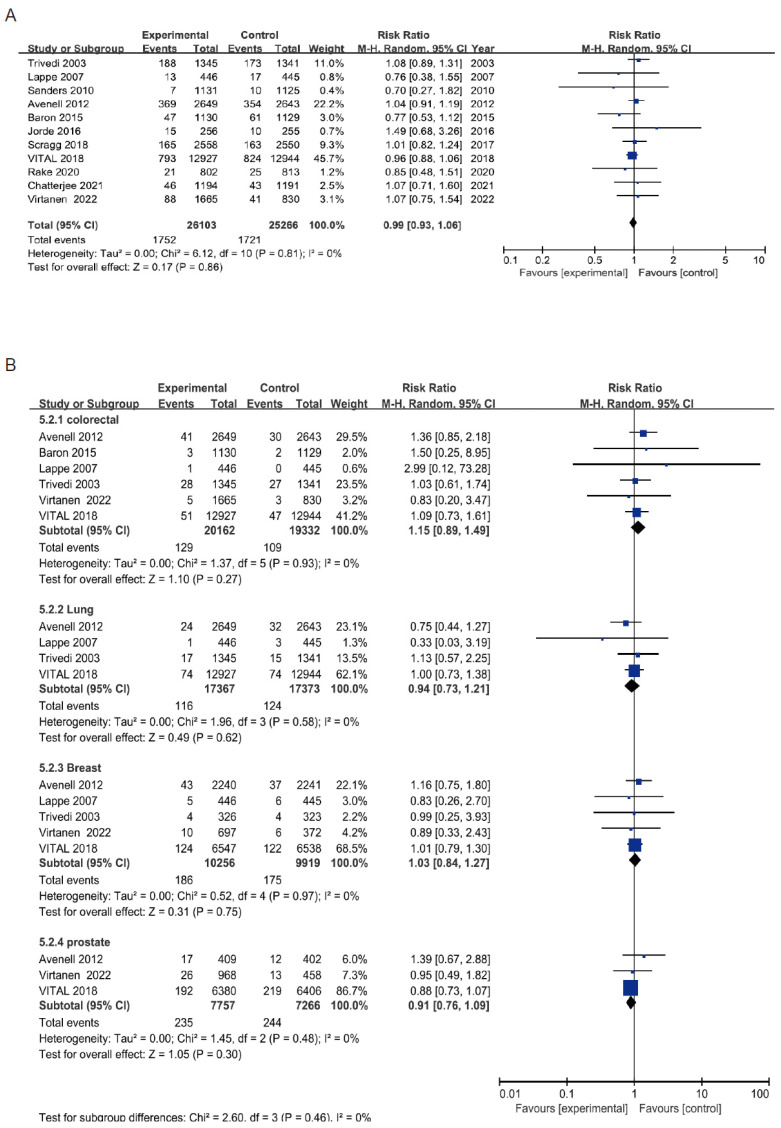
The forest plot of vitamin D supplementation and cancer incidence. (**A**) overall cancer incidence, (**B**) site-specific cancer incidence.

A total of 11 RCTs with a total of 51,369 participants were included in the meta-analysis for cancer incidence [14,30,32,34,35,37,38,41,42,43,45,46]. No significant association of vitamin D supplementation with overall cancer incidence was found (RR 0.99, 95% CI 0.93–1.06, I^2^ = 0%; Figure 4A). Similar results were also found in the analyses of site-specific cancer incidence, including lung, breast, prostate, and colorectal cancer (Figure 4B). TSA analysis showed that the pooled sample size was sufficient and further trials are unlikely to change the result for cancer incidence (Figure S4). The funnel plot, Egger and Begg’s tests showed no evidence of publication bias for the overall cancer incidence (Egger’s test: *p* = 0.78, Begg’s tests: *p* = 0.78, Figure S5).

### 3.4. Grading of Evidence

The GRADE summary findings for overall and site-specific cancer outcomes are shown in Table 2. The outcome of overall cancer mortality was found to be of moderate quality of evidence because of the inconsistency between studies, while the outcome of overall cancer incidence was deemed to be of high quality.

## 4. Discussion

The findings of our meta-analysis indicate that vitamin D supplementation does not reduce cancer mortality or incidence overall. For site-specific cancer outcomes, we found that vitamin D supplementation could reduce lung cancer mortality. Furthermore, only participants with daily dosing vitamin D have lower cancer mortality compared with those dosing vitamin D intermittently (RR 0.84, 95% CI 0.72–0.97).

Compared with early meta-analyses that included trials with mixed interventions of vitamin D supplementation combined with calcium supplementation [16,19,47], we did not include these trials in the present study because evidence showed calcium supplementation was associated with other unfavorable effects, including mortality [22], cardiovascular (e.g., myocardial infarction) [23,24,25], and breast cancer risk [26]. In addition, we did not include RCTs with a follow-up time of less than one year as 25(OH)D levels need 3 to 6 months to attain homeostasis after vitamin D supplementation and cancer mortality of less than one year is mostly due to undiagnosed metastasis of cancer at the start of study [16].

Our findings on cancer morality were inconsistent with the recent meta-analyses conducted [20,48]. The most recent meta-analysis conducted by Guo et al. found vitamin D supplementation to reduce cancer mortality (RR = 0.88, 95% CI 0.8 to 0.96) while our results found a null association (RR = 0.96, 95% CI 0.80 to 1.16; *p* = 0.68). The main inconsistency mainly came from the results of the D-Health Trial, which was published recently [15]. In the D-Health Trial, Neale et al. found that the vitamin D supplementation arm has, although statistically insignificant, higher cancer mortality than the control group with a median 5.7 years follow-up (hazard ratios 1.15, 95%CI 0.96 to 1.39; *p* = 0.13) [15]. Our subgroup analyses found that participants with daily dosing vitamin D have lower cancer mortality compared with those dosing vitamin D intermittently (RR 0.84, 95% CI 0.72–0.97), which might partly explain the difference between the results of the D-Health Trial (which used monthly dosing) and other large RCTs including VITAL and RECORD trial (which used daily dosing) [37,38]. The results were consistent with Keum et al., which also found daily dosing instead of intermittent dosing of vitamin D, could reduce total cancer mortality [20]. Daily vitamin D might be a more effective way to increase 25(OH)D than intermittent dosing [49]. In addition, according to our TSA analyses, under the assumption of 20% relative risk reduction to maintain an overall 5% risk of type I error and 80% power, the D-Health Trial was an essential update to previous results of meta-analyses which had been underpowered for cancer mortality. By adding the results of the D-Health trial, our present meta-analysis results have reached the required information size.

In the site-specific cancer analysis, we observed that vitamin D supplementation was associated with lower lung cancer mortality. These results were partly consistent with previous in vitro and in vivo studies, which have shown vitamin D could inhibit tumor growth, and diet-derived vitamin D might be a direct therapeutic agent in the EGFR-mutant lung cancer [50,51]. It has also been found that calcitriol, the active form of vitamin D, could inhibit lung cancer growth, metastases, and recurrence in mouse models [52,53]. Some epidemiological evidence, including a dose-response meta-analysis of prospective cohort studies, also supported our results that higher plasma 25-hydroxyvitamin D concentrations are associated with lower lung cancer mortality [54]. However, several studies have reported the opposite results, showing higher lung cancer mortality in participants with higher circulating 25-Hydroxyvitamin D [55,56] or a lack of difference [57,58]. Thus, our findings regarding lung cancer should be interpreted with caution because of the limited number of studies and sample size. Thus, further RCTs or large observational studies may be warranted.

We conducted the present review based on a protocol published in the PROSPERO database, which used a rigorous methodological approach based on the Cochrane Handbook. The strengths of this study included a rigorous assessment of the quality of evidence of included studies and the minimum information size was satisfied according to TSA.

Limitations should also be noted. First, our meta-analysis was based on published trials that reported cancer mortality. However, most trials of vitamin D supplementation did not include cancer mortality as an outcome, which might lead to bias of selective reporting. Second, the pooled sample size was large enough to evaluate the associations of vitamin D supplementation with total cancer mortality; however, the sample size is insufficient for specific subtypes of cancer. Additionally, studies included in our meta-analysis were highly purified compared with other meta-analyses, which may introduce additional bias.

## 5. Conclusions

The results of the current meta-analysis may have significant implications for clinicians and researchers. We suggest a reconsideration of the previous view that vitamin D supplementation could reduce overall cancer mortality. Different dosing frequencies might be necessary for future studies investigating the relationships between vitamin D supplementation and cancer mortality.

## Figures and Tables

**Table 1:** Characteristics of the 10 Randomized Clinical Trials.

Trial	Age (Years)	Female	Participants (Vitamin D/No Vitamin D)	Baseline 25OHD (nmol/L) (Vitamin D/No Vitamin D)	Intervention	Control	Primary Outcome	Follow-Up Period
Baron 2015 [43]	58	37%	1130/1129	58/58	vitamin D3 (1000 IU) daily plus calcium (1200 mg) daily	calcium (1200 mg) daily	adenomas incidence	3.7 years
Chatterjee 2021 [14]	60	44.5%	1194/1191	70/70	vitamin D3 4000 IU daily	placebo	cancer and major cardiovascular events	2.9 years
Jorde 2016 [35]	62	49%	256/255	60/61	vitamin D3 (20,000 IU) weekly	placebo	progression to type 2 diabetes	5 years
Lappe 2007 [30]	67	100%	446/445	72/72	vitamin D3 (1000 IU) plus calcium (1400 to 1500 mg) daily	calcium (1400 to 1500 mg) daily	fracture	4 years
Neale 2022 [15]	69	45.9%	10661/10649	NR	vitamin D3 60,000 IU monthly	placebo	mortality	5 years
Avenell 2012 [38]	77	85%	2649/2643	38/38	vitamin D3 (800 IU) or calcium (1000 mg) or both daily	calcium (1000 mg) or placebo daily	mortality	2–5.2 years with 3 follow-up years after intervention
Sanders 2010 [34]	76	100%	1131/1125	53/45	vitamin D3 (500,000 IU) yearly	placebo	falls and fractures	3–5 years with 1 follow-up years after intervention
Trivedi 2003 [41]	75	24%	1345/1341	NR	vitamin D3 (100,000 IU) four-monthly	placebo	Fracture	5 years
Scragg 2018 [32]	65.9	42%	2558/2550	64/63	vitamin D3 initial (200,000 IU) then vitamin D3 (100,000 IU) monthly	placebo	CVD and death	3.3 years
VITAL 2018 [37,42]	67	51%	12917/12944	77/77	vitamin D3 (2000 IU) daily	placebo	cancer and major cardiovascular events	5.3 years
Rake 2020 [45]	65–84 years	0.469	802/813	51.5/51.5	vitamin D3 (100,000 IU) monthly	placebo or no treatment	mortality	5 years
Virtanen 2022 [46]	68	0.428	830/1665	73/75	vitamin D3 (1600 IU or 3200 IU) daily	placebo	cardiovascular disease and cancer	5 years

**Table 2:** Summary of Findings and Strength of Evidence.

Outcome	No. of Patients (Trials)	RR (95%CI)	Absolute Effect Estimates (per 1000)			Quality of the Evidence
			Control	Intervention	Difference	
Cancer mortality	61882 (6)	0.96 (0.80 to 1.16)	22	21	−1 (−4 to 4)	Moderate
Colorectal cancer mortality	33849 (3)	0.91 (0.49 to 1.67)	2	2	0 (−1 to 2)	Moderate
Lung cancer mortality	29288 (3)	0.63 (0.45 to 0.90)	6	4	−2 (−1 to −3)	High
Breast cancer mortality	40132 (3)	1.04 (0.58 to 1.87)	1	1	0 (0 to 1)	Moderate
Prostate cancer mortality	26682 (2)	0.6 (0.21 to 1.74)	1	1	0 (−1 to 1)	Low
Cancer incidence	51369 (11)	0.99 (0.93 to 1.06)	68	67	−1 (−5 to 4)	High
Colorectal cancer incidence	39494 (6)	1.15 (0.89 to 1.49)	6	7	1 (−1 to 3)	High
Lung cancer incidence	34740 (4)	0.94 (0.73 to 1.21)	7	7	0 (−2 to 1)	High
Breast cancer incidence	20175 (5)	1.03 (0.84 to 1.27)	18	18	1 (−3 to 5)	High
Prostate cancer incidence	15023 (3)	0.91 (0.76 to 1.09)	34	33	−1 (−11 to 12)	High

## Supplementary Materials

The following supporting information can be downloaded at: https://www.mdpi.com/article/10.3390/cancers14153717/s1, Table S1: Search strategy; Table S2 Subgroup analysis of the effect of vitamin D on cancer mortality; Table S3: Sensitivity analyses of cancer mortality; Figure S1: Risk of bias summary: review authors’ judgements about each risk of bias item for each included study. Figure S2: Risk of bias graph: review authors’ judgements about each risk of bias item presented as percentages across all included studies; Figure S3. Funnel plot of cancer mortality; Figure S4. TSA analyses for cancer incidence; Figure S5. Funnel plot of cancer incidence.
