# Supplementary material for: Association between Vitamin D Supplementation and Cancer Mortality: A Systematic Review and Meta-Analysis
Source: Cancers (Basel). 2022 Jul 30;14(15):3717. doi: 10.3390/cancers14153717 (PMC9367315; doi:10.3390/cancers14153717)

Table S1: Search strategy

**MEDLINE(R)**		
1	exp Vitamin D/ad, ae, pd, tu [Administration & Dosage, Adverse Effects, Pharmacology, Therapeutic Use]	34315
2	((vit-d? or vitamin-d? or dihydroxyvitamin D? or dihydroxy-vitamin D? or hydroxyvitamin D? or hydroxyl-vitamin d? or dihydrotachysterol or colecalciferol or epicalcitriol or oxacalcitriol or alfacalcidol or calcifediol I or calciferol? or calcipotriol or calcitriol or dihydroxycolecalciferol or hydroxycolecalciferol or seocalcitol or tacalcitol or oxavitamin or hydroxycholecalciferol? or calcidiol or calcipotriene or dihydroxycholecalciferol? or dihydroxy-cholecalciferol or cholecalciferol? or ergocalciferol? or epiergocalciferol or dihydroxyergocalciferol or dihydroxy-ergocalciferol or hydroxyl-ergocalciferol or hydroxyergocalciferol or doxercalciferol or hydroxycalciferol or hydroxyl-calciferol or dihydroxy-calciferol or dihydroxycalciferol or dihydrotachysterin or calcamine or ercalcidiol) adj5 (supplement* or therap* or treat* or prevent* or daily or receiv* or regimen or dose? or oral* or intramuscular or inject*)).mp.	25508
3	randomized controlled trial.pt.	559804
4	controlled clinical trial.pt.	94698
5	random*.mp.	1351458
6	placebo.ab.	206921
7	drug therapy.fs.	2451261
8	trial.ab.	515291
9	groups.ab.	1992668
10	or/3-9	5111173
11	exp animals/ not humans.sh.	4966928
12	10 not 11	4380918
13	(1 or 2) and 12	19483
14	limit 13 to "all adult (22 plus years)"	10260
**Embase**		
1	exp vitamin D/ae, ct, ad, cb, cm, cr, do, dt, dl, du, ig, im, na, ut, iv, po, pa, pd, li, tp, td [Adverse Drug Reaction, Clinical Trial, Drug Administration, Drug Combination, Drug Comparison, Drug Concentration, Drug Dose, Drug Therapy, Intradermal Drug Administration, Intraduodenal Drug Administration, Intragastric Drug Administration, Intramuscular Drug Administration, Intranasal Drug	48473

	Administration, Intrauterine Drug Administration, Intravenous Drug Administration, Oral Drug Administration, Parenteral Drug Administration, Pharmacology, Sublingual Drug Administration, Topical Drug Administration, Transdermal Drug Administration]	
2	((vit-d? or vitamin-d? or dihydroxyvitamin D? or dihydroxy-vitamin D? or hydroxyvitamin D? or hydroxyl-vitamin d? or dihydrotachysterol or colecalciferol or epicalcitriol or oxacalcitriol or alfacalcidol or calcifediol I or calciferol? or calcipotriol or calcitriol or dihydroxycolecalciferol or hydroxycolecalciferol or seocalcitol or tacalcitol or oxavitamin or hydroxycholecalciferol? or calcidiol or calcipotriene or dihydroxycholecalciferol? or dihydroxy-cholecalciferol or cholecalciferol? or ergocalciferol? or epiergocalciferol or dihydroxyergocalciferol or dihydroxy-ergocalciferol or hydroxyl-ergocalciferol or hydroxyergocalciferol or doxercalciferol or hydroxycalciferol or hydroxyl-calciferol or dihydroxy-calciferol or dihydroxycalciferol or dihydrotachysterin or calcamine or ercalcidiol) adj5 (supplement* or therap* or treat* or prevent* or daily or receiv* or regimen or dose? or oral* or intramuscular or inject*)).mp.	46064
3	randomized controlled trial/	699449
4	crossover procedure/	69670
5	double blind procedure/	193122
6	single blind procedure/	45464
7	(random* or factorial* or crossover* or placebo* or assign* or allocat* or volunteer* or (doubl* adj5 blind*) or (singl* adj5 blind*)).mp.	2913659
8	or/3-7	2913659
9	exp animal/	28281020
10	human/	23222826
11	9 not 10	5058194
12	8 not 11	2638366
13	(1 or 2) and 12	16446
14	limit 13 to (adult <18 to 64 years> or aged <65+ years>)	7216

EBM Reviews - Cochrane Central Register of Controlled Trials

1	exp Vitamin D/ad, ae, aa, bi [Administration & Dosage, Adverse Effects, Analogs & Derivatives, Biosynthesis]	6
2	((vit-d? or vitamin-d? or dihydroxyvitamin D? or dihydroxy-vitamin D? or hydroxyvitamin D? or hydroxyl-vitamin d? or dihydrotachysterol or colecalciferol or epicalcitriol or oxacalcitriol or alfacalcidol or calcifediol I or calciferol? or calcipotriol or calcitriol or dihydroxycolecalciferol or hydroxycolecalciferol or seocalcitol or tacalcitol or oxavitamin or hydroxycholecalciferol? or calcidiol or calcipotriene or dihydroxycholecalciferol? or dihydroxy-cholecalciferol or cholecalciferol? or ergocalciferol? or epiergocalciferol or dihydroxyergocalciferol or dihydroxy-ergocalciferol or hydroxyl-ergocalciferol or	12299

	hydroxyergocalciferol or doxercalciferol or hydroxycalciferol or hydroxyl-calciferol or dihydroxy-calciferol or dihydroxycalciferol or dihydrotachysterin or calcamine or ercalcidiol) adj5 (supplement* or therap* or treat* or prevent* or daily or receiv* or regimen or dose? or oral* or intramuscular or inject*)).mp.	
3	1 or 2	12300

Table S2 Subgroup analysis of the effect of vitamin D on cancer mortality.

**Subgroup**	**Trials (patients)**	**RR, 95%CI**	**p**	**p for interaction**
**No. of patients**				0.03
≥2000	5(60267)	0.93 [0.80, 1.09]	0.37	
<2000	1(1615)	2.84 [1.03, 7.84]	0.04	
**No. of events**				0.52
≥200	3(52473)	0.93 [0.75, 1.16]	0.55	
<200	3(9409)	1.11 [0.69, 1.80]	0.67	
**Age**				0.96
≥70	3(9593)	0.97 [0.68, 1.40]	0.88	
<70	3(52289)	0.98 [0.77, 1.26]	0.89	
**Sex**				NA
Female	0(0)	NA	NA	
Both	6(61882)	0.96 [0.80, 1.16]	0.68	
**Baseline vitamin D status**				0.41
Adequacy (⩾50 nmol/L)	3(32594)	1.08 [0.66, 1.76]	0.77	
Insufficiency (<50 nmol/L)	1(5292)	0.85 [0.69, 1.04]	0.12	
Unknown	2(23996)	1.04 [0.80,1.35]	0.78	
**Published year**				0.22
Before 2014	2(7978)	0.85 [0.72, 1.02]	0.08	
In or after 2014	4(52289)	1.06 [0.79, 1.42]	0.70	
**Type of vitamin D**				NA
Vitamin D3	6(61882)	0.96 [0.80, 1.16]	0.68	
Vitamin D2	0(0)	NA	NA	
**Daily dose equivalent**				0.28
≥2000 IU	5(56590)	1.01 [0.80, 1.27]	0.96	
<2000 IU	1(5292)	0.85 [0.69, 1.04]	0.12	
**Timing**				0.09
Daily	2(31163)	0.84 [0.72, 0.97]	0.02	
Intermittently	4(30719)	1.09 [0.84, 1.42]	0.53	
**Treatment duration**				NA
duration ≥3 y	6(61882)	0.96 [0.80, 1.16]	0.68	
duration <3 y	0(0)	NA	NA	
**Co-therapy**				0.18
Without calcium	5(56590)	1.00 [0.88, 1.13]	0.97	
With calcium	1(5292)	0.84 [0.67, 1.05]	0.12	

Table S3: Sensitivity analyses of cancer mortality

**Sensitivity analyses**	**Risk Ratio, 95% CI**	**I^2**	**P**
Excluding studies with high or unknown risk of bias	0.98 [0.77,1.26]	63%	0.89
Excluding studies with high or unknown risk of bias of each domain			
Sequence generation	0.99 [0.79,1.23]	65%	0.90
Allocation concealment	0.94 [0.78,1.14]	58%	0.53
Blinding of patients and personnel	0.94 [0.78,1.14]	58%	0.53
Blinding of outcome assessors	0.94 [0.78,1.14]	58%	0.53
Incomplete outcome data	0.96 [0.80,1.16]	58%	0.68
Selective reporting	1.06 [0.79, 1.42]	68%	0.70
Other bias	0.96 [0.80, 1.16]	58%	0.68
Excluding quasi-randomized or cluster-randomized trials	0.93 [0.80, 1.09]	46%	0.37
Using fix-effect models	0.96 [0.86, 1.06]	58%	0.43
Excluding the largest trial	1.01 [0.81, 1.26]	57%	0.93

Figure S1: Risk of bias summary: review authors' judgements about each risk of bias item for each included study

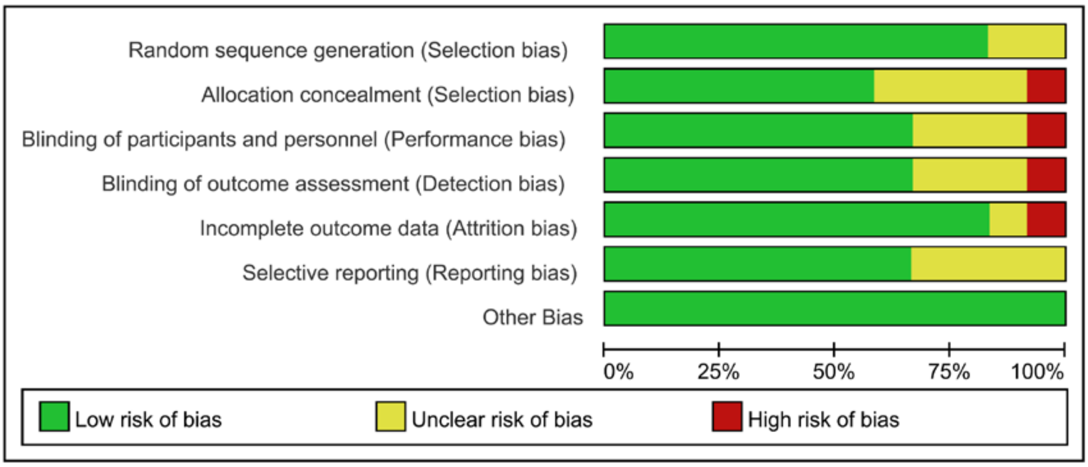

Figure S2: Risk of bias graph: review authors' judgements about each risk of bias item presented as percentages across all included studies

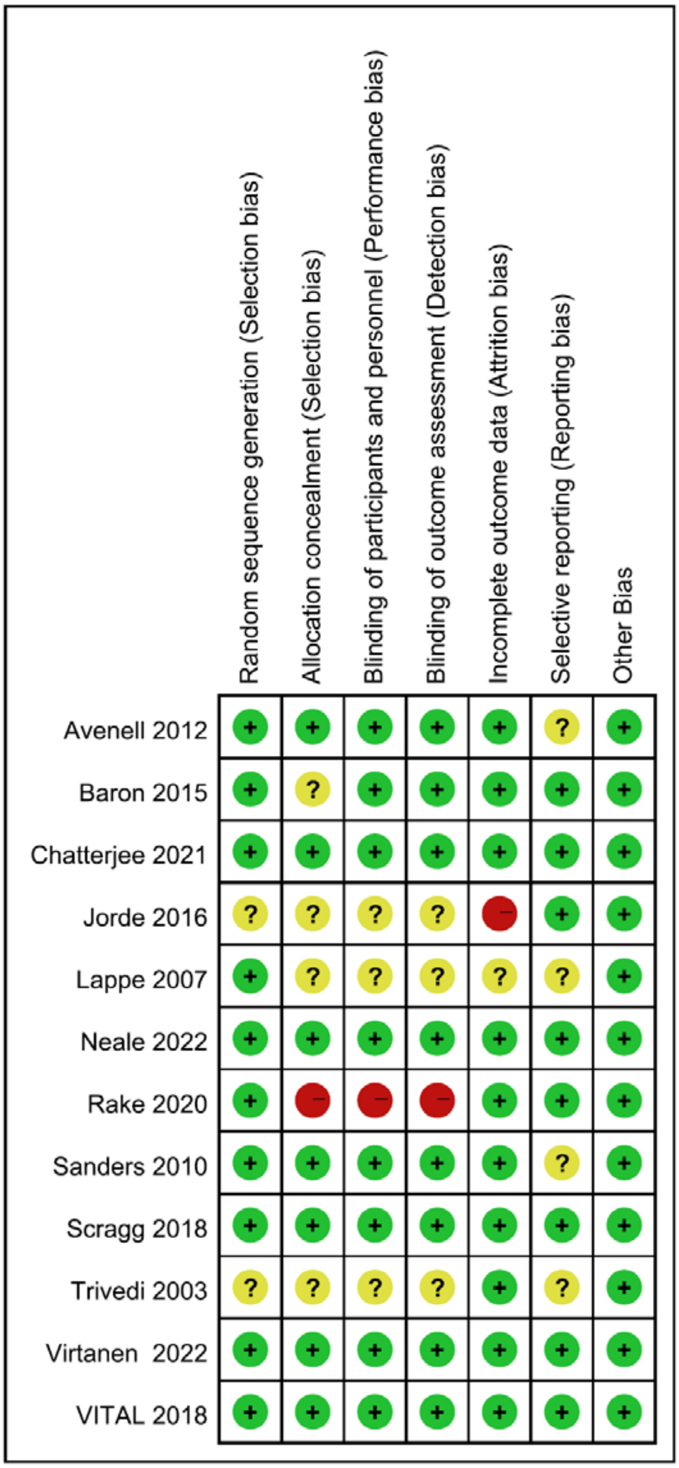

Figure S3. Funnel plot of cancer mortality

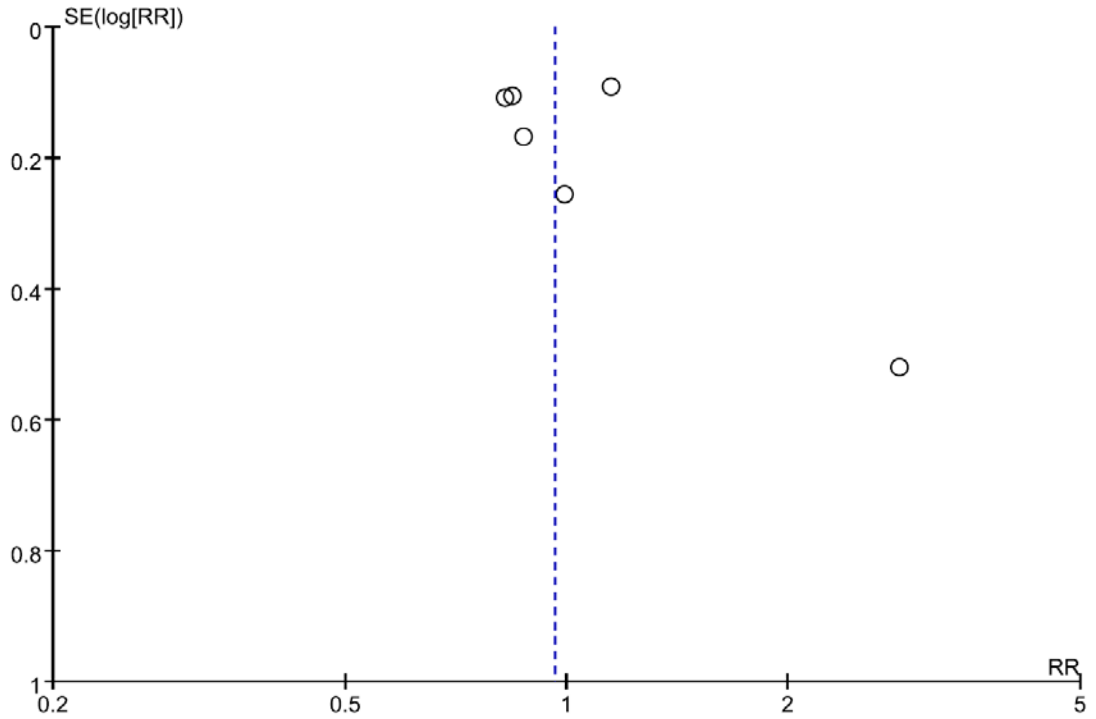

Figure S4. TSA analyses for cancer incidence.

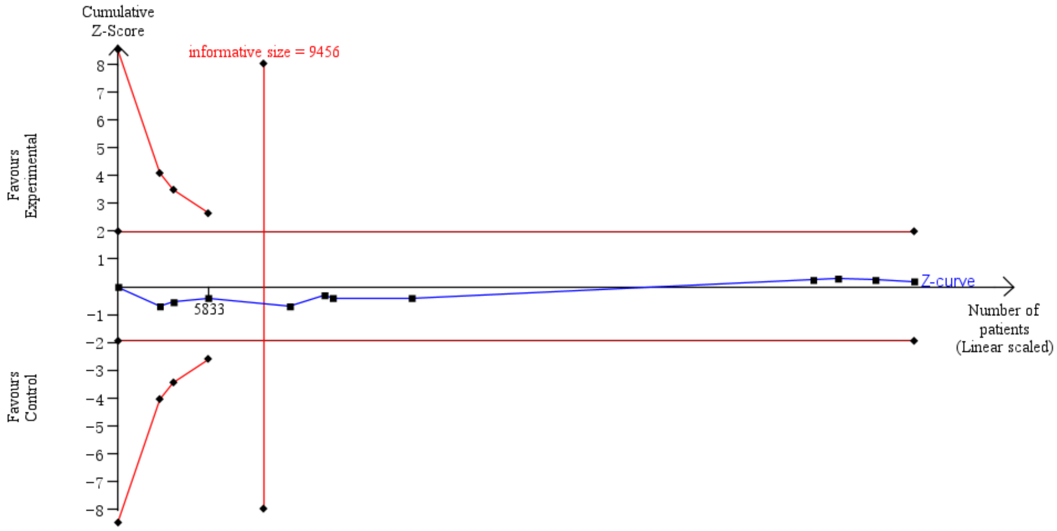

Figure S5. Funnel plot of cancer incidence

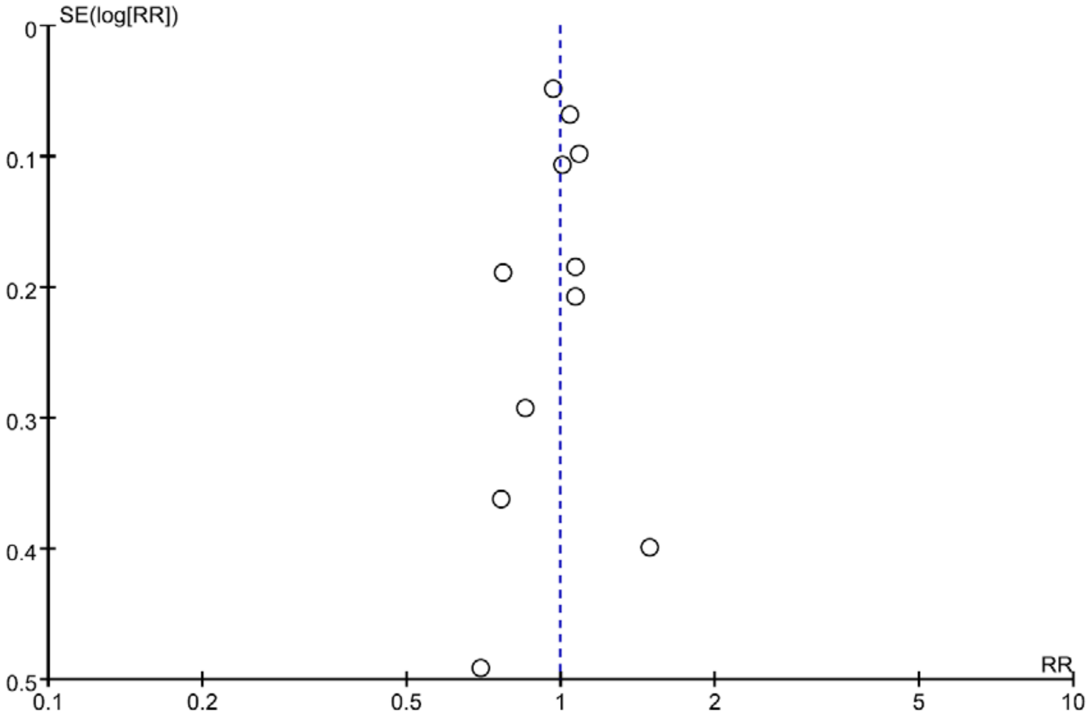

Supplement: Supplementary file 1 [file cancers-14-03717-s001.zip › 0722_Supplemental e-material_new.pdf]
